# Crystal Structure of the Full-Length Japanese Encephalitis Virus NS5 Reveals a Conserved Methyltransferase-Polymerase Interface

**DOI:** 10.1371/journal.ppat.1003549

**Published:** 2013-08-08

**Authors:** Guoliang Lu, Peng Gong

**Affiliations:** 1 State Key Laboratory of Virology, Wuhan Institute of Virology, Chinese Academy of Sciences, Wuchang District, Wuhan, Hubei, China; 2 University of Chinese Academy of Sciences, Beijing, China; The Scripps Research Institute, United States of America

## Abstract

The flavivirus NS5 harbors a methyltransferase (MTase) in its N-terminal ≈265 residues and an RNA-dependent RNA polymerase (RdRP) within the C-terminal part. One of the major interests and challenges in NS5 is to understand the interplay between RdRP and MTase as a unique natural fusion protein in viral genome replication and cap formation. Here, we report the first crystal structure of the full-length flavivirus NS5 from Japanese encephalitis virus. The structure completes the vision for polymerase motifs F and G, and depicts defined intra-molecular interactions between RdRP and MTase. Key hydrophobic residues in the RdRP-MTase interface are highly conserved in flaviviruses, indicating the biological relevance of the observed conformation. Our work paves the way for further dissection of the inter-regulations of the essential enzymatic activities of NS5 and exploration of possible other conformations of NS5 under different circumstances.

## Author Summary

Due to limited coding capacity, RNA viruses often generate proteins that contain more than one enzyme module to fulfill their rather complicated life cycle. Among those, the flavivirus nonstructural protein NS5 comprises an N-terminal methyltransferase (MTase) and a C-terminal RNA-dependent RNA polymerase (RdRP), playing key roles in processes including viral genome replication and capping. Although high-resolution crystal structures are available for MTase or RdRP alone, the intra-molecular interactions between the two modules remain elusive. By solving the crystal-structure of the full-length Japanese encephalitis virus NS5, we provide the first high-resolution readout of NS5 in its integrity, featuring an MTase-RdRP interface that is highly conserved in flaviviruses. Flaviviruses also include other important human pathogens such as dengue, West Nile, yellow fever, and tick-borne encephalitis viruses, currently lacking effective anti-viral drug. The conserved interface revealed by our structure thus may provide possibilities for the pharmaceutical community in the development of anti-flavivirus drug in a broad-spectrum manner.

## Introduction

As a genus of viruses in the family *Flaviviridae* among positive-strand RNA viruses, flaviviruses have more than 70 members, often causing human encephalitis and hemorrhagic diseases. Among these, the mosquito-borne species include important human pathogens such as dengue (DENV), yellow fever (YFV), West Nile (WNV), and Japanese encephalitis (JEV) viruses, affecting about one-third of the world population, mostly in tropical and subtropical regions. Currently, there is no effective antiviral drug available for all flaviviruses, and vaccines are lacking for DENV and WNV. The 10–11 kilo-base positive-sense flavivirus RNA genome contains both 5′ and 3′ untranslated regions (UTRs) and a single open reading frame that is translated into a large polyprotein. The genome bears a cap 1 structure (^N7Me^G5′-ppp-5′A_2′OMe_) at its 5′ end [1], while its 3′ end is not poly-adenylated. The polyprotein is processed into three structural and seven non-structural proteins. Among those, the ≈900-residue non-structural protein 5 (NS5) comprises an N-terminal S-adenosyl-L-methionine (SAM)-dependent methyltransferase (MTase) domain, and a C-terminal RNA-dependent RNA polymerase (RdRP) region that harbors the classic thumb, palm, and fingers domains present in all single-subunit polymerases. Although MTase is common for viruses bearing a 5′ cap structure and RdRP is required for all RNA viruses, the flavivirus NS5 represents a unique natural fusion of these two important enzymes. However, whether and how inter-regulations and cooperativity take place between the two enzymes of NS5 remain elusive.

Since 2002, the crystal structure of the ≈260-residue MTase domain has been reported in eight flaviviruses [2]–[4]. The flavivirus MTase is believed to catalyze both the N7 and 2′-O methylation steps, and may also act as a guanylyltransferase (GTase) to form the G5′-ppp-5′A linkage [5], [6], thereby playing key roles in the capping process. In 2007, the crystal structures of the RdRP region (the C-terminal 630 residues) from WNV and DENV were reported [7], [8]. Despite the fact that polymerase motifs F and G were not resolved, these RdRP structures exhibit high degree of similarity to the non-structural protein 5B (NS5B) from hepatitis C virus (HCV) and bovine viral diarrhea virus (BVDV) that represent the other two genera of the family *Flaviviridae* [9], [10]. Different from primer-dependent RdRPs such as poliovirus (PV) 3D [11], [12], these polymerases contain a “priming element” that partially occupies the putative ds-RNA channel (also termed “front channel”) and is believed to play an important role in *de novo* initiation of the RNA synthesis.

The MTase domain and the RdRP region are connected by a 10-residue linker (residues 266–275 in JEV NS5), and the crosstalk between these two parts of the NS5 has been reported [7], [13]. NS5 has also been documented to interact with other viral replication proteins including the non-structural protein 3 (NS3) protease/helicase, and to recruit promoter-like element stem loop A (SLA) in the 5′ region of the viral genome for precise initiation of RNA synthesis [14]. Moreover, NS5 is involved in the importin-mediated nuclear import and exportin CRM1-mediated nuclear export, through its nuclear localization signals (NLSs) and nuclear export sequence (NES), respectively [15]–[18]. However, the understanding of these NS5-related molecular interactions is greatly hindered by the lacking of a crystal structure of the full-length protein. Nevertheless, two structural models of the full-length NS5 have been proposed. The first model places the MTase domain off the RdRP ds-RNA channel, largely based on reverse genetics analysis [7], and the second model has the two regions loosely associated in a drastically different relative orientation, relying on small-angle X-ray scattering (SAXS) data [19]. With an aim to further the grip on NS5, we have crystallized the full-length JEV NS5 protein and solved a 2.6-Å resolution crystal structure. The structure provides the first high-resolution snapshot of the flavivirus NS5, elucidates conserved intra-molecular interactions between MTase and RdRP, and paves a way to dissect the versatile functions of NS5 in its natural form.

## Results

### Protein crystallization and structure determination

A C-terminal hexahistidine-tagged JEV full-length NS5 was purified to homogeneity and then subjected to crystallization trials. Small rod-like crystal was obtained in initial rounds of crystallization screening. The growth condition and strategy were optimized to yield crystals that are at least 100-micron in each dimension. The structure was solved through comprehensive molecular replacement trials using multiple search models derived from the flavivirus MTase and RdRP crystal structures [8], [20]. The unit cell contains three NS5 hexamers (trimer of dimers) in the H3 space group (Table 1), and the asymmetric unit comprises a NS5 dimer with the two molecules arranged in a pseudo two-fold symmetry (Fig. S1 A/B in Text S1). Despite the observation of the oligomeric forms in the crystal lattice, JEV NS5 primarily exists as a monomer in gel-filtration chromatography (Fig. S1C in Text S1).

**Table 1:** Data collection and refinement statistics.

	JEV NS5^a^
**Data collection**	
Space group	H3
Cell dimensions	
*a*, *b*, *c* (Å)	272.3, 272.3, 177.2
α,β, γ (°)	90, 90, 120
Resolution (Å)	46.2-2.60 (2.69-2.60)^b^
*R*_merge_	0.058 (0.356)
*I*/σ*I*	8.4 (1.6)
Completeness (%)	98.7 (97.0)
Redundancy	2.16 (2.12)
**Refinement**	
Resolution (Å)	2.60
No. of unique reflections	148758
*R*_work_/*R*_free_	0.196/0.228
No. atoms	
Protein	14288
Ligand/ion	74
Water	382
*B*-factors	
Protein	58.1
Ligand/ion	65.9^c^
Water	53.5
R.m.s. deviations	
Bond lengths (Å)	0.01
Bond angles (°)	1.16

a One crystal was used for this structure.

b Values in parentheses are for highest-resolution shell.

c Ligand/ion include four Zinc ions, two bound to each NS5 molecule, and two SAH molecules, one bound to each NS5 molecule.

### Overall structure of JEV NS5

**Figure 1:**
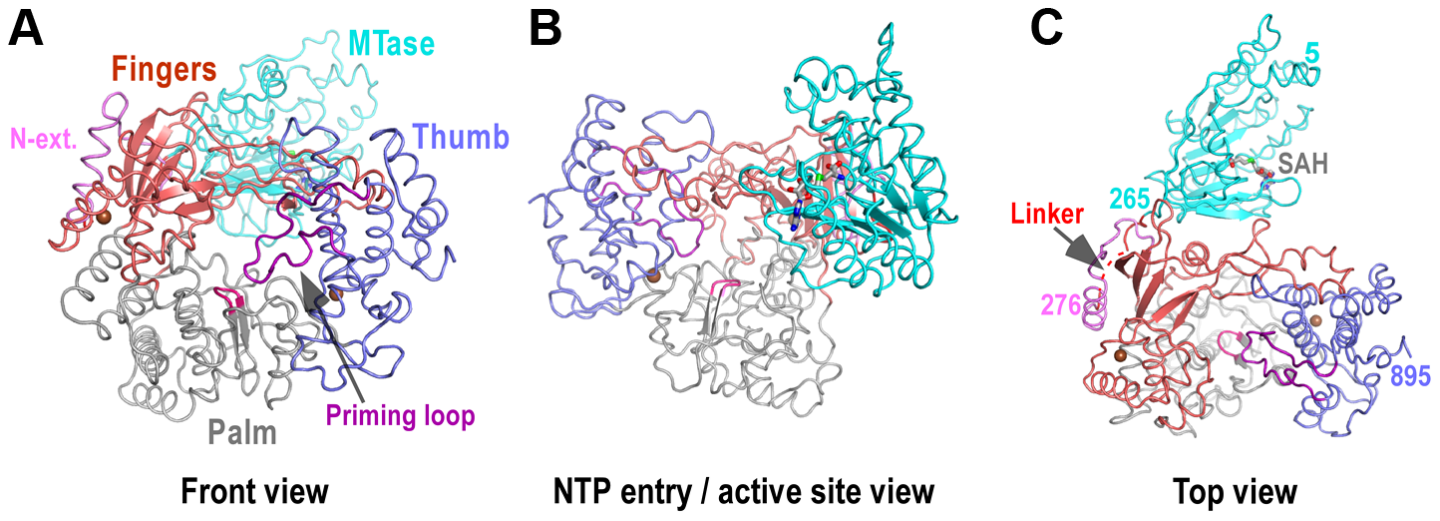
Global views of JEV NS5 structure. Structure of NS5 shown in orientations A) looking into the RdRP front channel (putative dsRNA channel); B) along the NTP entry channel; C) viewing from the top of RdRP. MTase is in cyan, RdRP palm in gray, thumb in blue, fingers in light red, N-terminal extension in pink, priming loop in purple, and signature sequence SGDD in magenta. Zinc ions are shown as brown spheres. The numbers defining the residue ranges of MTase and RdRP are shown in panel C. The three missing residues (271–273) in the linker are indicated by dashed lines in panel C. For clarity purposes, protein structures in all figures are shown in thin ribbon style with only the key β-sheet structures shown in regular cartoon style in most of the figure panels. “N-ext.” is used as abbreviation for “N-terminal extension”.

**Figure 2:**
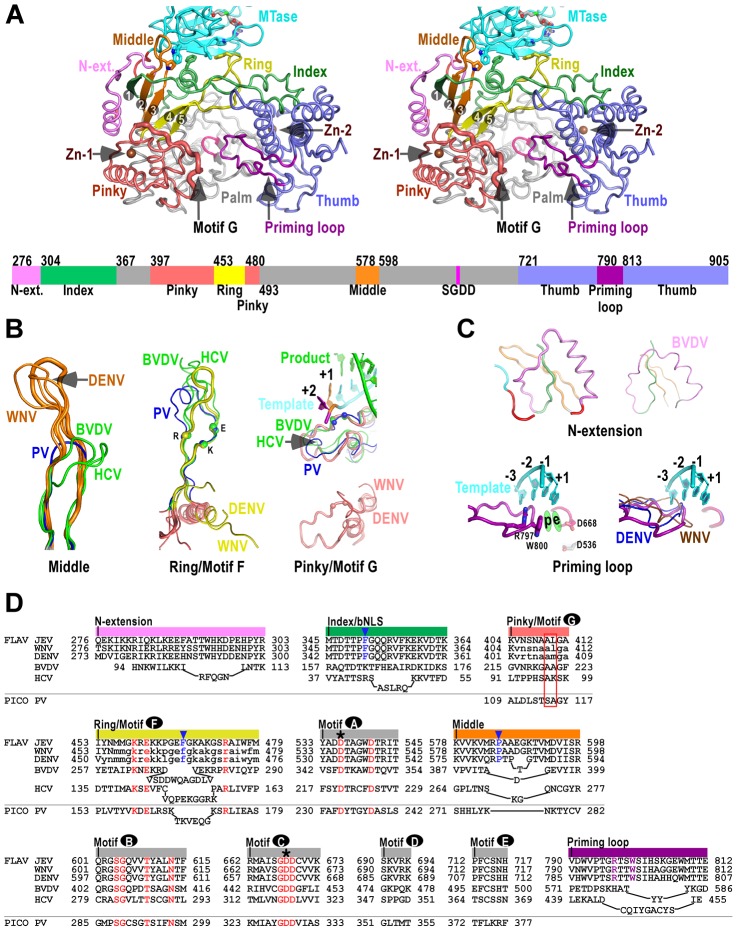
The RdRP region of flavivirus NS5. A) Stereo-pair images of NS5 viewing down into the RdRP active site (same view as in Fig. 1C) with a color-coded bar defining structural elements underneath. Coloring scheme is as in Figure 1, except that the index (green), middle (orange), ring (yellow), and pinky (light red) fingers are individually color-coded. Side chains of key residues in the MTase-RdRP interface are shown in sticks. The strand numbers of the 5-stranded β-sheet are indicated. B–C) Structural comparison of important elements in the core polymerase (B), the N-terminal extension and the priming loop (C). The JEV model is shown as thick ribbons and colored as in panel A. Three highly conserved charged residues in motif F (K459, E461, R474) and two structurally conserved motif G residues (A410, L411) interacting with the +1/+2 junction of the template strand are shown as spheres. The RNA duplex in the PV elongation complex (EC) model is shown in the motif G subpanel. The template strand in the priming loop subpanel is modeled using the PV EC model. Side chains of key priming loop residues W800, R797 and the invariant D536, D668 are shown in sticks. The putative priming NTP site (“p”) and the elongating NTP site (“e”) are indicated. D) Structure-based sequence alignment of RdRP motifs A–G, and other important elements. Three viruses from flavivirus genus, HCV and BVDV representing the other two genera of the *Flaviviridae* family, and PV representing viruses using primer-dependent strategy in genome replication are included in the alignment. Conserved active site residues (red text), MTase interacting residues (blue text and triangle), priming loop residues (purple text) are highlighted, the two invariant catalytic Asp residues are highlighted by asterisks, and residues in lower case letters either deviate from the consensus structure conformations or are not resolved in the crystal structures and are therefore included based only on sequence homology. The structurally conserved residues interacting with the template +1/+2 junction is highlighted by a red box, Colors at top of the alignment correspond to coloring of the structural elements in panel A. FLAV and PICO are used as abbreviations for *Flaviviridae* and *Picornaviridae* Families, respectively.

Only three small segments of NS5 are unresolved in the full-length structure: residues 1–4 and 896–905 at the N- and C-termini, respectively, and residues 271–273 in the MTase-RdRP linker (Fig. 1). The overall folds of MTase and RdRP are largely consistent with existing flavivirus NS5 crystal structures for individual enzymes (Figs. 1–2, Fig. S2A in Text S1). The MTase (residues 1–266) is complexed with the S-adenosyl-L-homocysteine (SAH), the demethylated form of SAM. Unlike previously reported RdRP structures, the RdRP region of NS5 is intact, with motifs F and G completely resolved. The linker is largely seen with only three residues (271–273) not modeled due to weakness of the electron density. The missing of residues 271–273 is not a result of proteolysis since the protein is intact in the crystal (Fig. S1D in Text S1). The distance between α-carbon atoms of residues 270 and 274 in the model is about 13 Å, at least 17 Å shorter than any other possible connections in the crystal lattice, thus making the current model unambiguous. Mediated by the linker, the MTase domain is attached to the backside of the RdRP through key hydrophobic interactions (details below), shielding the top-right rim of NTP entry channel (Fig. 1B).

### The RdRP region – core polymerase

The JEV NS5 RdRP region comprises a core polymerase, an N-terminal extension (residues 276–303), and a thumb domain insertion (residues 790–812, often termed “priming loop”) common to RdRPs that initiate RNA synthesis *de novo* (Fig. 2A). As with other viral RdRPs, the core polymerase adopts a shape analogous to a cupped right hand, with thumb and fingers rising on sides of the palm. The palm domain is the most conserved part of viral RdRPs, containing two catalytic aspartic acid residues (D536 of motif A and D668 of motif C in JEV NS5) that are absolutely conserved in all single-subunit processive polymerases [12]. The thumb domain of viral RdRP is relatively diverse. In general, RdRPs that initiates via *de novo* mechanism such as JEV NS5 have a bulkier thumb than those of primer-dependent RdRPs, carrying additional elements (either as insertions and/or C-terminal extensions) that facilitate *de novo* initiation [7]–[9], [21]. Since the structural features of the palm and thumb domains in the JEV NS5 are mostly consistent with the existing WNV and DENV RdRP models (Fig. S2A in Text S1), here we focus on illustration of the fingers domain of the JEV NS5 that has novel observations within.

**Figure 3:**
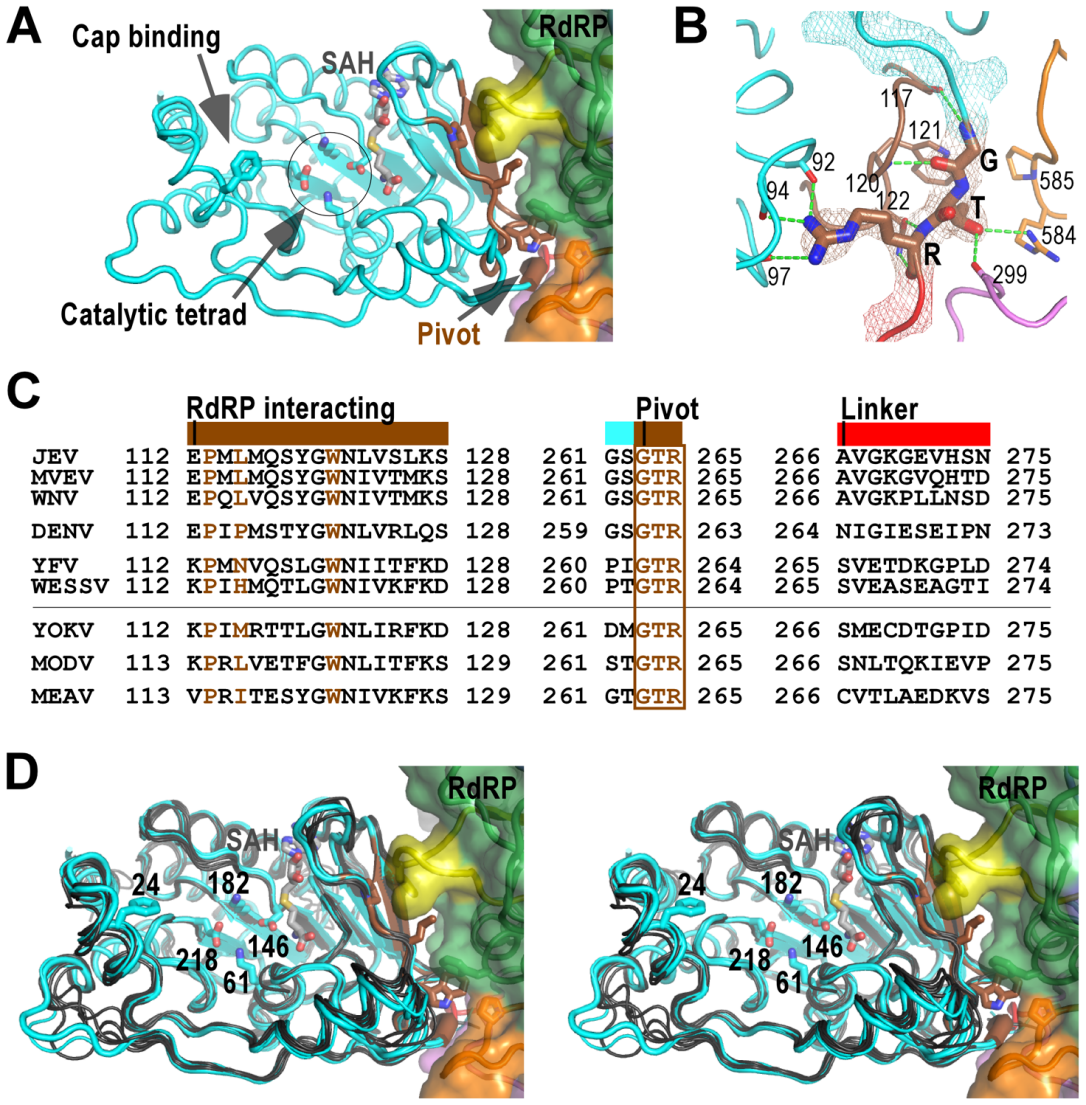
The MTase domain of flavivirus NS5. A) Structure of NS5 viewing into the MTase catalytic cleft. The RdRP interacting module (residues 112–128) and the hypothesized “pivot” region (residues GTR from 263 to 265) are shown in brown. The methyl donor derivative SAH, and the side chains of catalytic tetrad K-D-K-E, GTP/cap binding residue F24, and key hydrophobic residues in the MTase-RdRP interface are shown as sticks. Surface representation of RdRP is also shown. Color-coding is the same as in Figure 2 unless mentioned otherwise. B) Hydrogen bonding network (dashed lines) surrounding the GTR pivot region. The pivot is shown as sticks and with 3,500 K composite simulated-annealing (SA) omit electron density map (contoured at 1.5σ) overlaid. C) Structure-based sequence alignment of the RdRP interacting module, GTR pivot, and linker from nine flaviviruses. Three key residues (P113, L115, and W121) and the pivot residues GTR are highlighted in brown. Colors at top of the alignment correspond to coloring of the structural elements in panel A. All virus abbreviations used in the alignment are those of the International Committee on Taxonomy of Viruses. D) Structural conservation of the MTase among all flaviviruses shown by stereo images of superposition of MTase structures from nine viral species. JEV (thick), MVEV, and WNV MTase structures are shown in cyan. The other structures are shown in dark grey. Representations of JEV NS5 are as in panel A.

To better describe the NS5 RdRP structure and its interactions with the MTase, we define individual finger subdomains according to a nomenclature first used in PV RdRP [11] (Fig. 2A). The tip of the index finger interacts with the thumb domain, forming an encircled active site that is unique to viral RdRPs. The index finger also contains a nuclear localization signal βNLS (residues 322–370) that coincides with the suggested NES (residues 329–345) and NS3 binding site [15]–[17], [22], [23]. The highly conserved 20-residue core (residues 345–364) of this region centers at an α-helix that has been suggested to interact with importin β (Fig. 2D) [16]. The middle finger (residues 578–598) includes the second and third strands of the fingers domain 5-stranded β-sheet, and no specific function has been assigned to this element except for the contribution to the structural integrity of the fingers domain. Interestingly, the flavivirus RdRP middle finger is at least 7–8 residues longer than those of other positive-strand RNA virus RdRPs including HCV and BVDV NS5Bs (Fig. 2B/D), thus extruding to the surface of the protein. The ring finger (residues 453–479) includes the NTP binding motif F, containing the fourth and fifth strands of the 5-stranded β-sheet. In WNV and DENV RdRP structures, the majority of the ring finger is disordered and the authors proposed an alternate main chain path of this region. In our JEV NS5 structure, the ring finger is ordered and intact, forming the roof of the NTP entry channel, a canonical position observed in other viral RdRPs (Fig. 2A/B/D). It is noteworthy that the βNLS core helix of the index finger, the tip of the elongated middle finger, and the tip of the ring finger, line in a row at the top-right rim of the NTP entry channel, and interact with the MTase intra-molecularly through key hydrophobic interactions (Figs. 2–3, and details below). Comparing to other fingers, the pinky finger is relatively bulky and forms one side of the dsRNA channel. In primer-dependent viral RdRPs such as PV 3D^pol^, the pinky finger contains a conserved motif G (residues 109–118) that wedges at the +1/+2 kink of the template RNA, and its N-terminal half runs roughly parallel to the upstream template RNA (Fig. 2B) [12]. While the corresponding region is mostly disordered in the WNV and DENV RdRP structures, it is resolved in the JEV NS5 structure (residues 404–412), adopting a conformation consistent with that observed in HCV and BVDV NS5Bs (Figs. 2B, S2B). Although not conserved in sequence even within the *Flaviviridae* family, this region is structurally conserved among all viral RdRPs (Fig. 2B/D, Fig. S2B in Text S1), likely playing common roles in RdRP-RNA complex stability and/or RdRP translocation through its intimate association with the template strand.

Two Zn^2+^ binding sites have been identified in NS5 RdRP crystal structures. In the JEV NS5 structure, the Zn^2+^ bound to the pinky finger (Fig. 2A, Zn-1) has equivalent coordination partners (E440-H444-C449-C452) as previously observed, likely playing structural roles. The second Zn^2+^ is located between the thumb and motif E in the palm (Fig. 2A, Zn-2), a pivoting region that may be important for the commonly observed relative movement between thumb and palm [24]–[26], and might have regulatory roles to the polymerase as previously suggested [8]. It differs in one coordination partner comparing to that in the DENV structure (H717-C733-E848-C852 vs. H712-C728-H714-C847), while the WNV structure has a disulfide bond (C733-C852) at the same site [7], [8]. However, the functional consequences of these differences remain to be investigated.

### The RdRP region – the N-terminal extension and the priming loop

The N-terminal extension (residues 276–303) of the core polymerase was sometimes considered as part of the MTase. However, it associates with the core polymerase in the full-length context and has minimum interactions with the MTase domain (Fig. 2A). Interestingly, BVDV NS5B has an N-terminal stretch (residues 94–113) that is highly analogous in conformation [10], [27], albeit previously not noted (Fig. 3C/D). Although its function is unclear to date, the N-terminal extension may play auxiliary roles to RdRP by interacting with the first strand of the fingers domain 5-stranded β-sheet. Related to this, a WNV RdRP truncated at residue 317 has the entire middle finger, ring finger, and the majority of the index finger disordered in the crystal structure, and is catalytically inactive. In contrast, an RdRP truncated at residue 273 is active and has the index and middle finger properly folded with the N-terminal extension (Fig. 2A in Text S1) [7].

Viral RdRPs perform *de novo* RNA synthesis require an insertion in the thumb domain that projects into the active site, sometimes assisted by a C-terminal extension of the thumb [9], [10], [21]. As suggested in bacteriophage phi6 RdRP, by using an aromatic side chain (Trp in flaviviruses, Tyr in HCV and phi6) to set up a priming platform through stacking interactions to an initiating NTP, these elements play a critical role in the early stages of genome replication, and are believed to withdraw from the active site upon the growth of the template-product dsRNA during the transition to the elongation phase. The flavivirus NS5 has the priming element as a single loop structure (residues 790–812) connecting two α-helices of the thumb domain (Fig. 2A/C/D). In JEV NS5, Trp800 and Arg797 in this loop lined up through cation-π interactions [28] in an orientation suitable for priming the initiating NTP (Fig. 2C). In contrast to HCV and BVDV NS5B, the C-terminus of JEV NS5 is not a component of the priming platform, as also noted in the WNV RdRP structure [7]. Although the C-terminal 10 residues (896–905) are unresolved in the full-length structure, the nearly 40 Å distance between residues 895 and 800, and steric hindrance make it unlikely for the C-terminus to reach the vicinity of the active site.

### The MTase domain

The MTase domain adopts a canonical SAM-dependent methyltransferase fold with multiple helices flanking around a conserved 7-stranded β-sheet. The conformation of JEV MTase domain is largely consistent with the available MTase crystal structures from eight other flaviviruses (Fig. 3A). Amongst all nine viral species, Murray Valley encephalitis virus (MVEV), WNV, and JEV belong to the same group, and indeed their MTase structures exhibit highest similarity among all structures [20], [29]. The high degree of structural conservation also suggests that the MTase domain is quite rigid, not much affected by presence of its natural fusion partner RdRP.

Two regions in the MTase are of particular interest in the context of the full-length protein, and are neither spatially in proximity nor close in primary sequence to the MTase catalytic tetrad K61-D146-K182-E218. The first region, named RdRP interacting module herein, consists of residues 112–128, and plays a critical role in the intra-molecular interactions with the RdRP region (Fig. 3 and details below). The second region comprises the last three residues G263-T264-R265 of the MTase and is highly conserved in all flaviviruses (Fig. 3). In contrast, the linker region next to the GTR residues exhibits high degree of sequence variation (Fig. 3C). G263 and R265 form a total of seven hydrogen bonds with the rest of MTase, while T264 forms two hydrogen bonds with RdRP (Fig. 3B). Due to its critical location in the MTase-RdRP interface and critical position in linear sequence, we postulate that it may serve as a “pivot” in the establishment/disengagement of RdRP-MTase interactions observed in the full-length structure (Fig. 3 and discussed below).

### Interactions between MTase and RdRP

**Figure 4:**
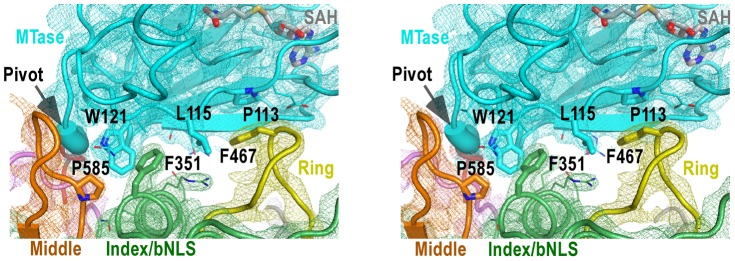
The MTase-RdRP interface. Stereo-pair images of 3,500 K composite SA-omit electron density map (contoured at 1.5σ) overlaid onto structural models centered at the MTase-RdRP interface. Coloring scheme is as in Figure 2A. The GTR pivot is shown as thick ribbon. Side chains of six key hydrophobic residues (thick) that form the interface core and other residues (thin) at the peripheral of the interface are shown in sticks.

Of the key findings in the full-length JEV NS5 structure, are the intra-molecular interactions between MTase and RdRP, with a total of 1480 Å^2^ buried in the interface. Although quite some polar/electrostatic interactions are present in the interface, the heart of the interactions is the formation of a hydrophobic network (Fig. 4). This involves residues Pro113, Leu115, and Trp121 from the MTase, and Phe467 (ring) Phe351 (index/βNLS core helix), and Pro585 (middle) from the RdRP fingers domain arranged in an alternating fashion (Figs. 2A, 3A, and 4). Notably, none of these six residues participates in catalysis, but five out of the six are highly conserved across the flavivirus genus, with the only exception being residue 115 that is also mostly hydrophobic (Fig. 3C), strongly implying the biological relevance of this observed conformation. Pro113/Leu115/Trp121 reside at the peripheral of the 7-stranded β-sheet that forms the base of the MTase catalytic platform. With the RdRP approaches almost perpendicular to the catalytic cleft, the SAM-binding, cap-binding, and 5′ RNA binding sites are fully accessible in this conformation (Fig. 3A).

There are three RdRP elements involved in the MTase-RdRP interface: the tip of the ring finger, the βNLS core helix of the index finger, and the tip of the elongated middle finger, each offering a conserved hydrophobic residue (Phe467, Phe351, Pro585, respectively) in the aforementioned hydrophobic network of the interface. Although quite some distance to the polymerase active site, the interactions with the MTase domain may modulate the movement of the fingers domain 5-stranded β-sheet that has been documented to undergo subtle rigid body movement upon active site closure for RdRP catalysis [12]. Beyond that, the presence of the MTase domain only partially shields the top-right corner of the NTP entry channel, leaving the template channel and dsRNA channel unaffected.

## Discussion

### A schematic of the full-length structure

The full-length JEV NS5 structure provides the first high-resolution readout of this multi-function protein essential for viral genome replication and RNA capping. It delineates all functional domains and key motifs of this 905-residue protein. The N-terminal 265-residue SAM-dependent MTase domain is highly analogous to all other flavivirus MTase crystal structures, which are unexceptionally solved with the core polymerase excluded from the constructs. To its C-terminus, the MTase is connected by a 10-residue linker (residues 266–275) whose sequence is highly variable among genus flavivirus (Fig. 3C). The linker is in turn connected to a 28-residue N-terminal extension (residues 276–303) of the core polymerase. This region is modestly conserved among flaviviruses and its function remains elusive. The core polymerase runs from residue 304 to the C-terminus of NS5, and adopts the canonical viral RdRP cupped right hand conformation with an encircled active site. All seven polymerase motifs (motifs A–G) common to viral RdRPs and reverse transcriptases are properly arranged around the polymerase active site, among which the fingers domain motifs F and G are properly resolved for the first time in genus flavivirus, while the motifs A–E are within the palm domain. The 23-residue priming loop (residues 790–812) project into the polymerase active site as an insertion in the thumb domain, playing a key role in *de novo* initiation process common to polymerases including those from *Flaviviridae* and *Cystoviridae* families [7], [21], [27], [30].

### The uniqueness of the MTase-RdRP interface

**Figure 5:**
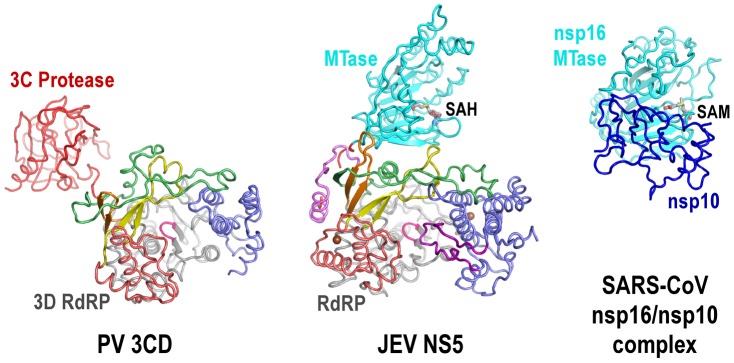
The uniqueness of the MTase-RdRP interface. Structures of JEV NS5 (middle) comparing to PV 3CD (left) and SARS-CoV nsp16/nsp10 complex (right). Viewing angle and coloring scheme are consistent with Figure 2A. The RdRP portion of PV 3CD is color-coded according to NS5 RdRP and protease is colored red. The SARS-CoV nsp16 MTase is shown in cyan and nsp16 stimulatory factor is shown in blue. The methyl donor SAM bound in nsp16 is shown as sticks.

The full-length JEV NS5 structure features a unique intra-molecular interface between the MTase and the RdRP. The PV 3CD protease is a precursor of the 3C protease and 3D polymerase. In its crystal structure, the protease domain connected to the RdRP region through a short linker, with minimal interactions between the two modules [31]. The spatial arrangement between RdRP and its N-terminal fused partner is also different. In NS5, MTase approaches the RdRP from the back, in a direction almost aligned with the dsRNA channel, while in 3CD the 3C protease resides at the left wing of the 3D polymerase backside (Fig. 5). In the crystal structure of the severe acute respiratory syndrome corona virus (SARS-CoV) nsp16/nsp10 binary complex, the stimulatory factor nsp10 forms extensive interactions with the nsp16 MTase. Different from the JEV NS5 that has the RdRP approaches almost perpendicularly to the opening of the MTase catalytic cleft, SARS-CoV nsp10 utilized a slightly concaved surface region to integrate itself to the SAM binding pocket and to extend the putative RNA binding groove of nsp16 MTase, thus enhancing the MTase activity [32]. In contrast, flavivirus MTase domain exhibits both the N7 and 2′-O methylation activity comparable to full-length NS5 in methylation assays *in vitro* [5], suggesting that the enzymatic function of the MTase is not much affected by its interactions with RdRP.

The MTase-RdRP interface is formed mainly by five elements, two from the MTase domain and three from the RdRP region. We define the first element in the MTase as RdRP interacting module (residues 112–128). It includes the β3 strand at the edge of the 7-stranded β-sheet and an extended β-like stretch antiparallel to β3. Among the three key MTase hydrophobic residues forming half of the interface core, Pro113 and Trp121 are highly conserved among known flaviviruses. The second element is the almost invariant GTR sequence at residues 263–265. Along with the spatially neighboring Trp121, the GTR sequence was hypothesized to mediate MTase-RdRP interactions due to its location at the terminus of the MTase [3]. On the RdRP side, the ring finger provides Phe467 at its tip to interact with Pro113/Leu115 of MTase, the βNLS core helix offers Phe351 to wedge between Leu115 and Trp121, and the elongated middle finger that is unique for flaviviruses participates this elegant hydrophobic network through Pro585. Together with the aforementioned key residues in the MTase domain, Phe467/Phe351/P585 are all highly and only conserved in flaviviruses, strongly arguing that the interactions observed in the JEV full-length structure is functionally relevant and common to the flavivirus genus.

### Possible other conformations of NS5

**Figure 6:**
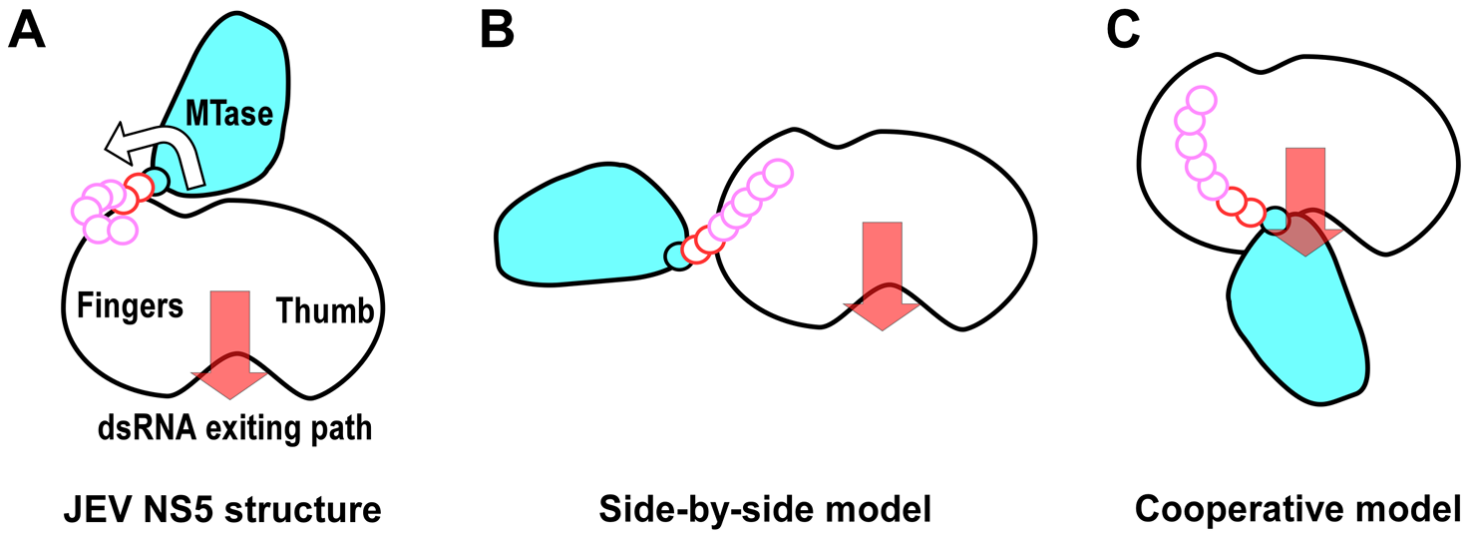
Cartoon illustration of possible conformations of NS5. The top view in Figure 1C was taken to sketch the JEV NS5 cartoon with MTase in cyan and the core polymerase in white. The GTR pivot (cyan), linker (red), and N-terminal extension (pink) are shown as continuous small circles. A) JEV NS5 model in this work. The hypothesized MTase disengagement from RdRP is depicted by the arrow. B) The elongated side-by-side model derived from the SAXS model of DENV NS5. C) The cooperative model adapted from the WNV NS5 model combining structures of individual MTase and RdRP regions and reverse genetics data from DENV.

The genome replication and capping processes involving flavivirus NS5 are both complicated with multiple distinct states. NS5 also interacts with other viral replication proteins, the 5′-SLA of the viral RNA genome, and cellular proteins that include transporters between the cytoplasm and the nucleus. Therefore, NS5 may adopt drastically different conformations under different circumstances. The βNLS of NS5 contains a highly conserved core (residues 345–364) centers around a helix that is largely occluded by the MTase domain in the full-length structure, making three β-importin interacting candidates R355/K358/K360 inaccessible. Together with the fact that the NS3 binding site was also mapped to the same region of βNLS, these observations argue that unraveling of the 1480 Å^2^ MTase-RdRP interface may occur if necessary. Among the two models of NS5 structure proposed previously, the 125 Å-long extended conformation suggested by SAXS data of DENV NS5 may reflect a form, rather than a defined conformation of NS5 with MTase and RdRP disengaged (Fig. 6B) [19]. The radius of gyration (R_g_) values of the JEV NS5 structure and the DENV NS5 SAXS model are 31.7 Å and ≈36 Å, respectively, indicating that these two models indeed differ in compactness. The model based on reverse genetics data places the MTase off the front channel (Fig. 6C) [7]. Such an arrangement would favor cooperativity of the two enzymes, but the validation of this cooperative model requires further structural evidence at high resolution.

Motifs F and G are responsible for NTP binding and template RNA binding, respectively. These two regions are both well resolved in the full-length JEV NS5 but disordered in WNV and DENV RdRP crystal structures. The formation of the MTase-RdRP interface likely makes motifs F and G less dynamic and helps maintain a canonical fold of RdRP, which in turn is beneficial for the initiation of RNA synthesis. Based on the spatial arrangement of the two key hydrophobic arrays P113/L115/W121 and F467/F351/P585, and the GTR pivot (Fig. 4), we postulate that NS5 could also open up the MTase-RdRP interface using GTR as the pivot to turn into a flexible state (Fig. 6 A–B) when interacting with other components of the genome replication machinery or transporters, or at certain stages of RNA replication. However, the existence of other defined conformation or the formation of an alternate interface between MTase and RdRP requires extra experimental evidences. The 10-residue linker not conserved in sequence would provide freedom for the sampling of other possible conformations to some extent, but larger scale rearrangements may require additional flexibility at the MTase-RdRP junction. Several evidences argue that the N-terminal extension (residues 276–303) may play a role in such a process. This 28-residue region is sometimes considered as part of the MTase [5], [33]. The only structural evidence supporting this came from the Wesselsbron virus MTase crystal structure that has the JEV 271–286 equivalent region folded back to the RdRP interacting module [34]. In contrast, all other crystal structures with this region resolved (including the JEV structure in this study) have it integrated to the core polymerase, wrapping around the first strand of the fingers domain 5-stranded β-sheet (Fig. 2A, Fig. S2A in Text S1). In addition, mutations within the C-terminal four residues 300–303 reduced the polymerase activity only to 40–70% [35], suggesting that the N-terminal extension could play auxiliary roles with respect to the polymerase. However, the association of the N-terminal extension with the RdRP may not be robust either. We have mentioned that BVDV NS5B residues 94–113 is structurally analogous to the N-terminal extension of JEV NS5. Although the same mode of interactions between the N-terminal extension and the fingers domain were observed, in the two crystal forms of the BVDV NS5B, one has the N-terminal extension folded with its own RdRP, and the other has it in a domain-swapping arrangement, associating with the RdRP from a second polypeptide chain. Taken together, the versatility of interactions offered by the N-terminal extension may significantly increase the spatial freedom for the placement of the MTase relative to the core polymerase, allowing the two regions to adopt drastically different relative orientations as needed for the sake of enzymatic activities, regulatory reasons, or MTase-RdRP cooperativity. For example, the conformation of the WNV NS5 cooperative model may be achieved with the flexibility of the N-terminal extension (depicted in Fig. 6C). Further efforts in obtaining more high-resolution NS5-containing structures are necessary to test this hypothesis.

### *De novo* RNA synthesis by viral RdRPs

The structure-function studies on primer-dependent viral RdRPs represented by PV 3D are greatly benefited from the fact that polymerase elongation complexes can be readily assembled using RNA construct with a template-primer duplex at least 6-basepair in length, so that the elongation process can be dissected independent of the naturally occurring complicated initiation process [12], [36]. In contrast, *de novo* viral RdRPs undergo an unstable initiation phase that is analogous to abortive cycling of DNA-dependent RNA polymerases and do not prefer primers longer than two nucleotides [37]–[41], making the *in vitro* assembly of either initiation complex or elongation complex very challenging for structural studies. One major advance in these RdRPs came from the crystal structure of the bacteriophage phi6 polymerase initiation complex obtained by co-crystallization, which started to reveal how these RdRPs utilize their own priming elements to facilitate initiation. Upon the transition to the elongation phase, the priming element of *de novo* RdRP is expected to withdraw from the active site and reach its destiny that is unknown to date. Recently, a co-crystal structure of HCV NS5B and end-protected template-primer RNA were obtained using a construct with a deletion in the priming element [42]. The structure doesn't resolve the remainder of the priming platform and very much resembles primer-dependent RdRP-RNA complexes [12], [25], [36]. In a separate study, very stable elongation complex was assembled using wild type HCV NS5B and a dinucleotide primer [43]. However, robust *in vitro* RdRP assays suitable for assembly of RdRP-RNA complex for structural studies have not been established in flavivirus NS5 to date. The full-length JEV NS5 structure presented here would set up an integral framework for such efforts. Together with recent advances in HCV NS5B, the molecular details of RNA synthesis by *de novo* RdRPs have begun to unravel.

In summary, the JEV NS5 crystal structure has provided a comprehensive view of this multi-function enzyme that is essential in viral RNA replication and capping processes. The structure reveals that the MTase attaches to the backside of the RdRP through interactions that are conserved and unique for the whole flavivirus genus. Implications and hypothesis regarding possible other functional conformations were discussed considering available structural and functional studies. The structure sets up the stages to explore functional polymerase-RNA complexes in different stages of RNA synthesis and to study the interactions between NS5 and viral/host factors, while the observation of the interface between the RdRP and MTase generates opportunities for antiviral development for JEV and other important human pathogens in flaviviruses.

## Materials and Methods

### Cloning and protein expression

The JEV NS5 gene was cloned into the pET26b-Ub-HCV-NS5B-Δ8 plasmid. The resulting plasmid, pET26b-Ub-JEV-NS5, was transformed into *Escherichia coli* strain BL21(DE3) pCG1 for expression where NS5 was initially produced as an ubiquitin fusion protein that was cleaved *in vivo* by a coexpressed ubiquitin-specific carboxyl terminal protease Ubp1 to produce full-length NS5 with the native N-terminal residue [44] and a C-terminal GSSS-His×6 tag. Cells were grown at 31.5°C overnight in NZCYM medium with 50 µg/ml kanamycin (KAN), 20 µg/ml chloramphenicol (CHL), and 0.5% (w/v) D-glucose until the OD_600_ was 1.0. The overnight culture was used to inoculate 1 L of NZCYM medium with 50 µg/ml KAN, 20 µg/ml CHL to reach an initial OD_600_ around 0.025. The cells were grown at 37°C at 250 rpm to an OD_600_ of 1.0 and then cooled to room temperature (r.t.). Isopropyl-β-D-thiogalactopyranoside (IPTG) was added to a final concentration of 0.5 mM, and the cells were grown for an additional 4 h before harvesting.

### Purification of JEV NS5

The cells were resuspended in a Lysis buffer of 300 mM NaCl, 50 mM Tris pH 8.0, 10 mM imidazole, 0.02% (w/v) NaN_3_, 20% (v/v) glycerol, and were lysed by passage through an AH-2010 homogenizer at 14,500 psi (ATS Engineering Ltd.). IGEPAL CA-630 (Sigma-Aldrich) was then added to a final concentration of 0.1% (v/v), and polyethylenimine (PEI) was then added slowly to 0.05% (v/v) over a 20-min period to precipitate nucleic acid. The lysate was slowly stirred at 4°C for an additional 15 min and then centrifuged for 40 min at 17,000 rpm in a SS-34 rotor (Thermo Scientific). The clarified lysate was loaded onto a nickel-charged HisTrap HP column (GE Healthcare), followed by step elution with 300 mM imidazole in 50 mM Tris pH 8.0, 300 mM NaCl, 20% (v/v) glycerol, and 0.02% (w/v) NaN_3_. Fractions containing NS5 were pooled and diluted to reduce the NaCl concentration to approximately 90 mM prior to loading onto a HiTrap SP HP column (GE Healthcare) and eluting with a linear gradient to 1 M NaCl in 25 mM MES pH 6.0, 0.1 mM EDTA, 20% (v/v) glycerol, and 0.02% (w/v) NaN_3_. The pooled fractions were concentrated to approximately 0.9 ml and run over a Superdex 200 gel filtration column (GE Healthcare) equilibrated in a GF buffer of 300 mM NaCl, 5 mM MES (pH 5.8), 20% (v/v) glycerol and 0.02% (w/v) NaN_3_. Pooled fractions were supplemented with tris-(2-carboxyethyl)phosphine (TCEP) to a final concentration of 5 mM, concentrated to approximately 15 mg/mL, flash frozen with liquid nitrogen, and stored at −80°C in 10–20 µl aliquots. The extinction coefficient of 220,615 M^−1^cm^−1^ was calculated based on protein sequence using the ExPASy ProtParam program (http://www.expasy.ch/tools/protparam.html). The typical yield is 3–5 mg of pure protein per liter of bacterial culture.

### Crystallization, data collection, and structure determination

Hexagonal prism-shaped crystals of JEV NS5 were obtained within 2 weeks by sitting drop vapor diffusion at 16°C using 8–10 mg/mL protein. Typically, a volume of 0.6–1 ul of protein solution at a concentration of 10 mg/ml in the GF buffer with 5 mM TCEP was mixed with an equal volume of a precipitant/well solution of 0.085 M trisodium citrate pH 5.6, 0.17 M potassium/sodium tartrate, 1.7 M ammonium sulfate, 15% (v/v) glycerol. Crystals were directly frozen and stored in liquid nitrogen prior to data collection. Initial data sets used for molecular replacement trials were collected at the Beijing Synchrotron Radiation Facility (BSRF) 3W1A beamline (wavelength = 1 Å, temperature = 100K). The final data set was collected at the Shanghai Synchrotron Radiation Facility (SSRF) beamline BL17U1 (wavelength = 0.9793 Å, temperature = 100K). At least ninety degrees of data were typically collected in 0.3° oscillation steps. Reflections were integrated, merged, and scaled using D*Trek v9.9 [45] with resulting statistics being listed in Table 1. The initial structure solution was obtained using the molecular replacement program PHASER [46]. Three search models were used in a sequential manner in a comprehensive molecular replacement trial: the MVEV MTase (pdb entry: 2PX2), and two parts of the DENV RdRP (pdb entry: 2J7U). Manual model rebuilding was performed using Coot [47] and refined with the PHENIX software suite [48]. Noncrystallographic (NCS) symmetry was applied to both chains in the asymmetric unit for MTase and RdRP separately in initial rounds of refinement and was released in later rounds. The Ramachandran statistics are 92.2%, 7.5%, 0.2%, and 0.1% for favored, allowed, generously allowed, and disfavored regions, respectively. The 3,500 K composite simulated-annealing (SA) omit electron density maps were generated by the program CNS [49] and structure figures were generated with PyMOL (www.pymol.org).

### Superpositioning of structures

Superimpositions shown in Figures 2A, 3D, S1B, S2A were performed using the maximum likelihood based multiple structure superpositioning program THESEUS (www.theseus3d.org). All other superimpositions were done using traditional least-square method with the following JEV-equivalent residues selected: 576–581 (Fig. 2B, middle finger subpanel); 453–460 (Fig. 2B, ring finger subpanel); 405–411 (Fig. 2B, motif G subpanel); 304–310 (Fig. 2C, N-terminal extension subpanel); 664–671 (Fig. 2C, priming loop subpanel, JEV superimpositions with BVDV, HCV, PV structures in Fig. S2B in Text S1, JEV superimposition with PV structure in Fig. 5); 76–80, 141–147, and 171–182 (the three central strands of the 7-stranded β-sheet of MTase; JEV superimposition with SARS-CoV structure in Fig. 5). The pdb entries used in the above superimpositions with JEV NS5 are: 2PX2 (MVEV MTase), 1L9K (DENV MTase), 2OY0 (WNV MTase), 3EVA (YFV MTase), 3ELD (WESSV MTase), 3GCZ (YOKV MTase), 2WA1 (MODV MTase), 2OXT (MEAV MTase), 2HFZ (WNV RdRP), 2J7U (DENV RdRP), 1S4F (BVDV NS5B), 1NB7 (HCV NS5B in complex with DNA), 3OL6 (PV 3D elongation complex), 3VWS (DENV RdRP with inhibitor).

### Accession number

Coordinates and structure factor files have been deposited in the Protein Data Bank, with accession code 4K6M.

## Supporting Information

Text S1 Figure S1. JEV NS5 forms a dimer in the asymmetric unit and is monomeric in solution. A) Surface representation of the NS5 dimer in the asymmetric unit viewing down the pseudo 2-fold axis. Coloring scheme: molecule I MTase - lime, RdRP - green; molecule II MTase - cyan, RdRP - blue; linker - grey. B) Maximum likelihood superimposition of the two NS5 molecules in the asymmetric unit (RMSD = 0.5 Å) shown as cartoon representation. Coloring scheme is as in panel A. C) NS5 has a retention volume around 68 mL in a superdex 200 gel filtration column, consistent with a monomeric state. Empirical retention volumes and molecular weights of three other globular proteins were indicated. PV and YFV are abbreviations of poliovirus and yellow fever virus, respectively. D) 10% SDS-PAGE analysis of the NS5 crystal. Lane 1: Washed crystal; lane 2: NS5 sample used for crystallization; lane 3: Empty; lane 4: Molecular weight marker. Figure S2. Structural comparisons of flavivirus RdRPs. A) Side by side comparison derived from a maximum likelihood superimposition of RdRP structures from JEV, WNV, and DENV, clearly showing the incompleteness and misfolding of motif F (yellow) and motif G (light red) in the latter two structures. Representations, viewing angle, and coloring scheme is as in the main text Figure 2A. The RMSD values of 559 structurally equivalent α-carbon atoms are 2.3 Å for JEV and WNV RdRPs and 1.4 Å for JEV and DENV RdRPs. The corresponding RMSD values for palm, thumb, and fingers domains individually are 1.0 Å/0.7 Å, 1.0 Å/0.6 Å, and 2.5 Å/1.8 Å (JEV and WNV/JEV and DENV), respectively. B) Comparison of RdRP motif G conformation of JEV, BVDV, HCV, showing that motif G of JEV NS5 adopt a canonical conformation. In a very recent report, binding of an inhibitor helped resolve the missing part of motif G in the original DENV RdRP model. However, the observed conformation deviates significantly from the JEV model.
(PDF)

## References

[1] Cleaves GR, Dubin DT (1979) Methylation status of intracellular dengue type 2 40 S RNA. Virology 96: 159–165. 111410 10.1016/0042-6822(79)90181-8

[2] Egloff MP, Benarroch D, Selisko B, Romette JL, Canard B (2002) An RNA cap (nucleoside-2′-O-)-methyltransferase in the flavivirus RNA polymerase NS5: crystal structure and functional characterization. EMBO J 21: 2757–2768. 12032088 10.1093/emboj/21.11.2757 PMC125380

[3] Liu L, Dong H, Chen H, Zhang J, Ling H, et al (2010) Flavivirus RNA cap methyltransferase: structure, function, and inhibition. Front Biol 5: 286–303. 10.1007/s11515-010-0660-y PMC3172701 21927615

[4] Geiss BJ, Thompson AA, Andrews AJ, Sons RL, Gari HH, et al (2009) Analysis of flavivirus NS5 methyltransferase cap binding. J Mol Biol 385: 1643–1654. 19101564 10.1016/j.jmb.2008.11.058 PMC2680092

[5] Ray D, Shah A, Tilgner M, Guo Y, Zhao Y, et al (2006) West Nile virus 5′-cap structure is formed by sequential guanine N-7 and ribose 2′-O methylations by nonstructural protein 5. J Virol 80: 8362–8370. 16912287 10.1128/JVI.00814-06 PMC1563844

[6] Issur M, Geiss BJ, Bougie I, Picard-Jean F, Despins S, et al (2009) The flavivirus NS5 protein is a true RNA guanylyltransferase that catalyzes a two-step reaction to form the RNA cap structure. RNA 15: 2340–2350. 19850911 10.1261/rna.1609709 PMC2779676

[7] Malet H, Egloff MP, Selisko B, Butcher RE, Wright PJ, et al (2007) Crystal structure of the RNA polymerase domain of the West Nile virus non-structural protein 5. J Biol Chem 282: 10678–10689. 17287213 10.1074/jbc.M607273200

[8] Yap TL, Xu T, Chen YL, Malet H, Egloff MP, et al (2007) Crystal structure of the dengue virus RNA-dependent RNA polymerase catalytic domain at 1.85-angstrom resolution. J Virol 81: 4753–4765. 17301146 10.1128/JVI.02283-06 PMC1900186

[9] Lesburg CA, Cable MB, Ferrari E, Hong Z, Mannarino AF, et al (1999) Crystal structure of the RNA-dependent RNA polymerase from hepatitis C virus reveals a fully encircled active site. Nat Struct Biol 6: 937–943. 10504728 10.1038/13305

[10] Choi KH, Groarke JM, Young DC, Kuhn RJ, Smith JL, et al (2004) The structure of the RNA-dependent RNA polymerase from bovine viral diarrhea virus establishes the role of GTP in de novo initiation. Proc Natl Acad Sci U S A 101: 4425–4430. 15070734 10.1073/pnas.0400660101 PMC384763

[11] Thompson AA, Peersen OB (2004) Structural basis for proteolysis-dependent activation of the poliovirus RNA-dependent RNA polymerase. EMBO J 23: 3462–3471. 15306852 10.1038/sj.emboj.7600357 PMC516629

[12] Gong P, Peersen OB (2010) Structural basis for active site closure by the poliovirus RNA-dependent RNA polymerase. Proc Natl Acad Sci U S A 107: 22505–22510. 21148772 10.1073/pnas.1007626107 PMC3012486

[13] Zhang B, Dong H, Zhou Y, Shi PY (2008) Genetic interactions among the West Nile virus methyltransferase, the RNA-dependent RNA polymerase, and the 5′ stem-loop of genomic RNA. J Virol 82: 7047–7058. 18448528 10.1128/JVI.00654-08 PMC2446981

[14] Filomatori CV, Lodeiro MF, Alvarez DE, Samsa MM, Pietrasanta L, et al (2006) A 5′ RNA element promotes dengue virus RNA synthesis on a circular genome. Genes Dev 20: 2238–2249. 16882970 10.1101/gad.1444206 PMC1553207

[15] Rawlinson SM, Pryor MJ, Wright PJ, Jans DA (2009) CRM1-mediated nuclear export of dengue virus RNA polymerase NS5 modulates interleukin-8 induction and virus production. J Biol Chem 284: 15589–15597. 19297323 10.1074/jbc.M808271200 PMC2708855

[16] Brooks AJ, Johansson M, John AV, Xu Y, Jans DA, et al (2002) The interdomain region of dengue NS5 protein that binds to the viral helicase NS3 contains independently functional importin beta 1 and importin alpha/beta-recognized nuclear localization signals. J Biol Chem 277: 36399–36407. 12105224 10.1074/jbc.M204977200

[17] Vasudevan SG, Johansson M, Brooks AJ, Llewellyn LE, Jans DA (2001) Characterisation of inter- and intra-molecular interactions of the dengue virus RNA dependent RNA polymerase as potential drug targets. Farmaco 56: 33–36. 11347963 10.1016/s0014-827x(01)01014-x

[18] Forwood JK, Brooks A, Briggs LJ, Xiao CY, Jans DA, et al (1999) The 37-amino-acid interdomain of dengue virus NS5 protein contains a functional NLS and inhibitory CK2 site. Biochem Biophys Res Commun 257: 731–737. 10208852 10.1006/bbrc.1999.0370

[19] Bussetta C, Choi KH (2012) Dengue virus nonstructural protein 5 adopts multiple conformations in solution. Biochemistry 51: 5921–5931. 22757685 10.1021/bi300406n PMC3448003

[20] Assenberg R, Ren J, Verma A, Walter TS, Alderton D, et al (2007) Crystal structure of the Murray Valley encephalitis virus NS5 methyltransferase domain in complex with cap analogues. J Gen Virol 88: 2228–2236. 17622627 10.1099/vir.0.82757-0

[21] Butcher SJ, Grimes JM, Makeyev EV, Bamford DH, Stuart DI (2001) A mechanism for initiating RNA-dependent RNA polymerization. Nature 410: 235–240. 11242087 10.1038/35065653

[22] Johansson M, Brooks AJ, Jans DA, Vasudevan SG (2001) A small region of the dengue virus-encoded RNA-dependent RNA polymerase, NS5, confers interaction with both the nuclear transport receptor importin-beta and the viral helicase, NS3. J Gen Virol 82: 735–745. 11257177 10.1099/0022-1317-82-4-735

[23] Pryor MJ, Rawlinson SM, Butcher RE, Barton CL, Waterhouse TA, et al (2007) Nuclear localization of dengue virus nonstructural protein 5 through its importin alpha/beta-recognized nuclear localization sequences is integral to viral infection. Traffic 8: 795–807. 17537211 10.1111/j.1600-0854.2007.00579.x

[24] Wu Y, Lou Z, Miao Y, Yu Y, Dong H, et al (2010) Structures of EV71 RNA-dependent RNA polymerase in complex with substrate and analogue provide a drug target against the hand-foot-and-mouth disease pandemic in China. Protein Cell 1: 491–500. 21203964 10.1007/s13238-010-0061-7 PMC4875138

[25] Zamyatkin DF, Parra F, Alonso JM, Harki DA, Peterson BR, et al (2008) Structural insights into mechanisms of catalysis and inhibition in Norwalk virus polymerase. J Biol Chem 283: 7705–7712. 18184655 10.1074/jbc.M709563200

[26] Jager J, Smerdon SJ, Wang J, Boisvert DC, Steitz TA (1994) Comparison of three different crystal forms shows HIV-1 reverse transcriptase displays an internal swivel motion. Structure 2: 869–876. 7529124 10.1016/s0969-2126(94)00087-5

[27] Choi KH, Gallei A, Becher P, Rossmann MG (2006) The structure of bovine viral diarrhea virus RNA-dependent RNA polymerase and its amino-terminal domain. Structure 14: 1107–1113. 16843892 10.1016/j.str.2006.05.020

[28] Brocchieri L, Karlin S (1994) Geometry of interplanar residue contacts in protein structures. Proc Natl Acad Sci U S A 91: 9297–9301. 7937759 10.1073/pnas.91.20.9297 PMC44799

[29] Zhou Y, Ray D, Zhao Y, Dong H, Ren S, et al (2007) Structure and function of flavivirus NS5 methyltransferase. J Virol 81: 3891–3903. 17267492 10.1128/JVI.02704-06 PMC1866096

[30] Bressanelli S, Tomei L, Roussel A, Incitti I, Vitale RL, et al (1999) Crystal structure of the RNA-dependent RNA polymerase of hepatitis C virus. Proc Natl Acad Sci U S A 96: 13034–13039. 10557268 10.1073/pnas.96.23.13034 PMC23895

[31] Marcotte LL, Wass AB, Gohara DW, Pathak HB, Arnold JJ, et al (2007) Crystal structure of poliovirus 3CD protein: virally encoded protease and precursor to the RNA-dependent RNA polymerase. J Virol 81: 3583–3596. 17251299 10.1128/JVI.02306-06 PMC1866080

[32] Chen Y, Su C, Ke M, Jin X, Xu L, et al (2011) Biochemical and structural insights into the mechanisms of SARS coronavirus RNA ribose 2′-O-methylation by nsp16/nsp10 protein complex. PLoS Pathog 7: e1002294. 22022266 10.1371/journal.ppat.1002294 PMC3192843

[33] Mastrangelo E, Bollati M, Milani M, Selisko B, Peyrane F, et al (2007) Structural bases for substrate recognition and activity in Meaban virus nucleoside-2′-O-methyltransferase. Protein Sci 16: 1133–1145. 17473012 10.1110/ps.072758107 PMC2206675

[34] Bollati M, Milani M, Mastrangelo E, Ricagno S, Tedeschi G, et al (2009) Recognition of RNA cap in the Wesselsbron virus NS5 methyltransferase domain: implications for RNA-capping mechanisms in Flavivirus. J Mol Biol 385: 140–152. 18976670 10.1016/j.jmb.2008.10.028

[35] Wang Q, Weng L, Tian X, Counor D, Sun J, et al (2012) Effect of the methyltransferase domain of Japanese encephalitis virus NS5 on the polymerase activity. Biochim Biophys Acta 1819: 411–418. 22285573 10.1016/j.bbagrm.2012.01.003

[36] Gong P, Kortus MG, Nix JC, Davis RE, Peersen OB (2013) Structures of coxsackievirus, rhinovirus, and poliovirus polymerase elongation complexes solved by engineering RNA mediated crystal contacts. PLoS One 8: e60272. 23667424 10.1371/journal.pone.0060272 PMC3648537

[37] Martin CT, Muller DK, Coleman JE (1988) Processivity in early stages of transcription by T7 RNA polymerase. Biochemistry 27: 3966–3974. 3415967 10.1021/bi00411a012

[38] Carpousis AJ, Gralla JD (1980) Cycling of ribonucleic acid polymerase to produce oligonucleotides during initiation in vitro at the lac UV5 promoter. Biochemistry 19: 3245–3253. 6996702 10.1021/bi00555a023

[39] Kao CC, Yang X, Kline A, Wang QM, Barket D, et al (2000) Template requirements for RNA synthesis by a recombinant hepatitis C virus RNA-dependent RNA polymerase. J Virol 74: 11121–11128. 11070008 10.1128/jvi.74.23.11121-11128.2000 PMC113194

[40] Selisko B, Dutartre H, Guillemot JC, Debarnot C, Benarroch D, et al (2006) Comparative mechanistic studies of de novo RNA synthesis by flavivirus RNA-dependent RNA polymerases. Virology 351: 145–158. 16631221 10.1016/j.virol.2006.03.026

[41] Gong P, Martin CT (2006) Mechanism of instability in abortive cycling by T7 RNA polymerase. J Biol Chem 281: 23533–23544. 16790422 10.1074/jbc.M604023200

[42] Mosley RT, Edwards TE, Murakami E, Lam AM, Grice RL, et al (2012) Structure of hepatitis C virus polymerase in complex with primer-template RNA. J Virol 86: 6503–6511. 22496223 10.1128/JVI.00386-12 PMC3393583

[43] Jin Z, Leveque V, Ma H, Johnson KA, Klumpp K (2012) Assembly, purification, and pre-steady-state kinetic analysis of active RNA-dependent RNA polymerase elongation complex. J Biol Chem 287: 10674–10683. 22303022 10.1074/jbc.M111.325530 PMC3323022

[44] Gohara DW, Ha CS, Kumar S, Ghosh B, Arnold JJ, et al (1999) Production of “authentic” poliovirus RNA-dependent RNA polymerase (3D(pol)) by ubiquitin-protease-mediated cleavage in Escherichia coli. Protein Expr Purif 17: 128–138. 10497078 10.1006/prep.1999.1100

[45] Pflugrath JW (1999) The finer things in X-ray diffraction data collection. Acta Crystallogr D Biol Crystallogr 55: 1718–1725. 10531521 10.1107/s090744499900935x

[46] McCoy AJ, Grosse-Kunstleve RW, Adams PD, Winn MD, Storoni LC, et al (2007) Phaser crystallographic software. J Appl Crystallogr 40: 658–674. 19461840 10.1107/S0021889807021206 PMC2483472

[47] Emsley P, Cowtan K (2004) Coot: model-building tools for molecular graphics. Acta Crystallogr D Biol Crystallogr 60: 2126–2132. 15572765 10.1107/S0907444904019158

[48] Adams PD, Grosse-Kunstleve RW, Hung LW, Ioerger TR, McCoy AJ, et al (2002) PHENIX: building new software for automated crystallographic structure determination. Acta Crystallogr D Biol Crystallogr 58: 1948–1954. 12393927 10.1107/s0907444902016657

[49] Brunger AT, Adams PD, Clore GM, DeLano WL, Gros P, et al (1998) Crystallography & NMR system: A new software suite for macromolecular structure determination. Acta Crystallogr D Biol Crystallogr 54: 905–921. 9757107 10.1107/s0907444998003254

