# Supplementary material for: Crystal Structure of the Full-Length Japanese Encephalitis Virus NS5 Reveals a Conserved Methyltransferase-Polymerase Interface
Source: PLoS Pathog. 2013 Aug 8;9(8):e1003549. doi: 10.1371/journal.ppat.1003549 (PMC3738499; doi:10.1371/journal.ppat.1003549)
Supplement: Text S1 — Figure S1. JEV NS5 forms a dimer in the asymmetric unit and is monomeric in solution. A) Surface representation of the NS5 dimer in the asymmetric unit viewing down the pseudo 2-fold axis. Coloring scheme: molecule I MTase - lime, RdRP - green; molecule II MTase - cyan, RdRP - blue; linker - grey. B) Maximum likelihood superimposition of the two NS5 molecules in the asymmetric unit (RMSD = 0.5 Å) shown as cartoon representation. Coloring scheme is as in panel A. C) NS5 has a retention volume around 68 mL in a superdex 200 gel filtration column, consistent with a monomeric state. Empirical retention volumes and molecular weights of three other globular proteins were indicated. PV and YFV are abbreviations of poliovirus and yellow fever virus, respectively. D) 10% SDS-PAGE analysis of the NS5 crystal. Lane 1: Washed crystal; lane 2: NS5 sample used for crystallization; lane 3: Empty; lane 4: Molecular weight marker. Figure S2. Structural comparisons of flavivirus RdRPs. A) Side by side comparison derived from a maximum likelihood superimposition of RdRP structures from JEV, WNV, and DENV, clearly showing the incompleteness and misfolding of motif F (yellow) and motif G (light red) in the latter two structures. Representations, viewing angle, and coloring scheme is as in the main text Figure 2A. The RMSD values of 559 structurally equivalent α-carbon atoms are 2.3 Å for JEV and WNV RdRPs and 1.4 Å for JEV and DENV RdRPs. The corresponding RMSD values for palm, thumb, and fingers domains individually are 1.0 Å/0.7 Å, 1.0 Å/0.6 Å, and 2.5 Å/1.8 Å (JEV and WNV/JEV and DENV), respectively. B) Comparison of RdRP motif G conformation of JEV, BVDV, HCV, showing that motif G of JEV NS5 adopt a canonical conformation. In a very recent report, binding of an inhibitor helped resolve the missing part of motif G in the original DENV RdRP model. However, the observed conformation deviates significantly from the JEV model. (PDF) [file ppat.1003549.s001.pdf]

Supporting Information

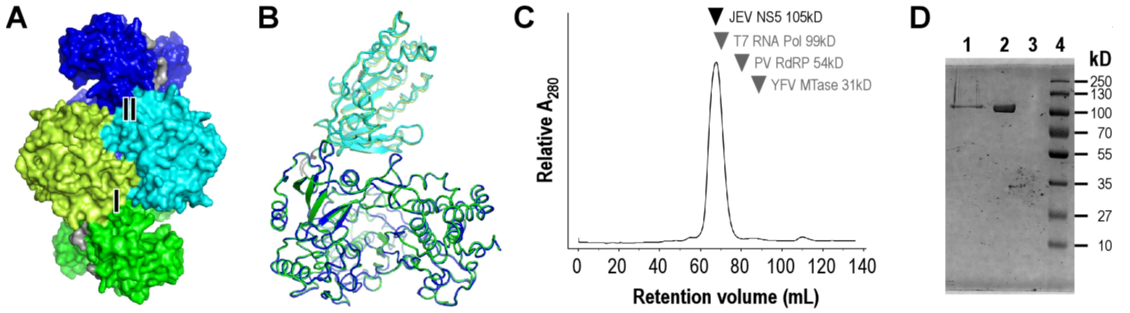

Supplementary Figure 1. JEV NS5 forms a dimer in the asymmetric unit and is monomeric in solution. A) Surface representation of the NS5 dimer in the asymmetric unit viewing down the pseudo 2-fold axis. Coloring scheme: molecule I MTase - lime, RdRP - green; molecule II MTase - cyan, RdRP - blue; linker - grey. B) Maximum likelihood superimposition [1] of the two NS5 molecules in the asymmetric unit (RMSD=0.5 Å) shown as cartoon representation. Coloring scheme is as in panel A. C) NS5 has a retention volume around 68 mL in a superdex 200 gel filtration column, consistent with a monomeric state. Empirical retention volumes and molecular weights of three other globular proteins were indicated. PV and YFV are abbreviations of poliovirus and yellow fever virus, respectively. D) 10% SDS-PAGE analysis of the NS5 crystal. Lane 1: Washed crystal; lane 2: NS5 sample used for crystallization; lane 3: Empty; lane 4: Molecular weight marker.

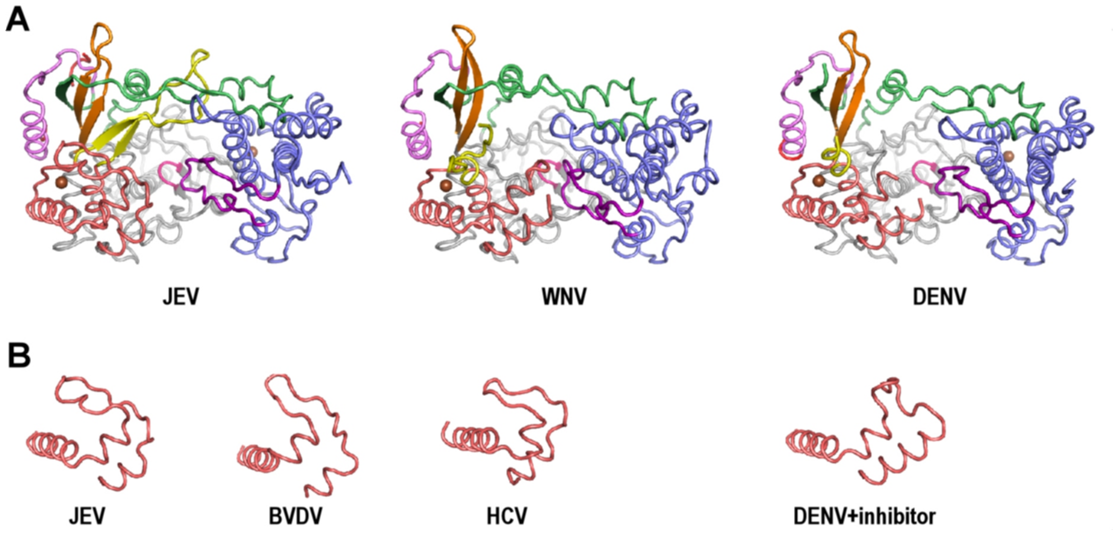

Supplementary Figure 2. Structural comparisons of flavivirus RdRPs. A) Side by side comparison derived from a maximum likelihood superimposition of RdRP structures from JEV, WNV, and DENV, clearly showing the incompleteness and misfolding of motif F (yellow) and motif G (light red) in the latter two structures. Representations, viewing angle, and coloring scheme is as in the main text Figure 2A. The RMSD values of 559 structurally equivalent α-carbon atoms are 2.3 Å for JEV and WNV RdRPs and 1.4 Å for JEV and DENV RdRPs. The corresponding RMSD values for palm, thumb, and fingers domains individually are 1.0 Å /0.7 Å, 1.0 Å /0.6 Å, and 2.5 Å /1.8 Å (JEV and WNV/JEV and DENV), respectively. B) Comparison of RdRP motif G conformation of JEV, BVDV, HCV, showing that motif G of JEV NS5 adopt a canonical conformation. In a very recent report [2], binding of an inhibitor helped resolve the missing part of motif G in the original DENV RdRP model. However, the observed conformation deviates significantly from the JEV model.

Supplementary References

1. Theobald DL, Wuttke DS (2006) THESEUS: maximum likelihood superpositioning and analysis of macromolecular structures. Bioinformatics 22: 2171-2172.
2. Noble CG, Lim SP, Chen YL, Liew CW, Yap L, et al. (2013) Conformational Flexibility of the Dengue Virus RNA-Dependent RNA Polymerase Revealed by a Complex with an Inhibitor. J Virol 87: 5291-5295.
